# Asylum-Seeking Children with Medical Complexity and Rare Diseases in a Tertiary Hospital in Switzerland

**DOI:** 10.1007/s10903-020-01100-8

**Published:** 2020-10-20

**Authors:** S. Buser, J. Brandenberger, M. Gmünder, C. Pohl, N. Ritz

**Affiliations:** 1 Migrant Health Service, University of Basel Children’s Hospital, Spitalstrasse 33, 4031 Basel, Switzerland; 2 Pediatric Emergency Department, University Children’s Hospital, Inselspital, University of Bern, Bern, Switzerland; 3 Neonatal Intensive Care Unit, Perth Children’s and King Edward Memorial Hospitals, Perth, Australia; 4 Paediatric Infectious Disease and Vaccinology, University of Basel Children’s Hospital, Basel, Switzerland; 5 Department of Paediatrics, Royal Children’s Hospital Melbourne, University of Melbourne, Parkville, Australia

**Keywords:** Chronic diseases, Europe, Genetics, Migrant health, Refugee minors

## Abstract

The aim of this study was to assess the characteristics of asylum-seeking children with medical complexity visiting a tertiary care hospital in Switzerland, detailing their underlying medical conditions and management. Asylum-seeking patients with frequent visits between January 2016 and December 2017 were identified using administrative and electronic health records. Of 462 patients, 19 (4%) fulfilled the inclusion criteria with 811 (45%) visits. The age of the 19 patients ranged from 0 to 16.7 years (median of 7 years) with two main age groups identified: < 2 years and > 12 years. Nine (47%) patients originated from Syria. A total of 34/811(4%) visits were hospital admissions, 66/811 (8%) emergency department visits and 320/811(39%) outpatient department visits. In children < 2 years genetic diseases (5/8; 63%) and nutritional problems (6/8; 75%) were most common; in adolescents, orthopedic diseases (4/8; 50%) and mental health problems (4/8; 50%). Asylum-seeking children with medical complexity represent a small but important group of patients requiring frequent medical consultations. The high proportion of young patients with genetic diseases and severe nutritional problems suggests that new strategies are required in the management of this specific group of asylum-seeking children. This could be achieved by improved co-ordination between hospital and non-hospital care exploring options for integrated care.

**Electronic supplementary material:**

The online version of this article (10.1007/s10903-020-01100-8) contains supplementary material, which is available to authorized users.

## Background

At the end of 2016 an estimated 65.6 million people were forcibly displaced worldwide, half of whom were children [1]. In the European Union 1.3 million people applied for asylum in 2016. One third of these asylum applicants were minor refugees below 18 years of age [2]. In Switzerland, an estimated 45,300 asylum applications were received in 2016 and 2017 accounting for 2% of all applications in Europe. Of those, an estimated 20,000 asylum applications were from children, including 2700 unaccompanied minor refugees [3].

Refugees may face specific health problems and challenges accessing healthcare in their place of origin, during the journey, and at their final destination [4]. There exists increasing concern about the health of refugees resulting in strategies and action plans for refugee and migrant health in Europe. According to the World Health Organization (WHO), host countries need to adapt their national health systems according to the challenges that refugees may face. This adaptation includes the improvement of collection of health-related data from asylum seekers to identify specific health needs [5].

Several studies have investigated the health status and health needs of asylum-seeking children visiting tertiary care hospitals [6–8]. These studies generally focus on the prevalence of communicable diseases and common problems and neglect to highlight the existence of asylum-seeking children with rare diseases needing complex care. A study from our institution describing the overall epidemiology and use of health care of asylum-seeking children showed that a small proportion of patients was responsible for nearly half of the total amount of visits [8]. A case-series from Germany including six asylum-seeking children with inborn metabolic diseases suggests that there is a need to be aware of genetic and complex chronic diseases in asylum-seeking children [9].

In recent years, research on children with special health care needs, a term defined by Maternal and Child Health Bureau in 1998 [10], has gained attention. One important subgroup are children with medical complexity defined as children with complex chronic conditions in need of frequent health care visits. This includes all children and adolescents with serious chronic conditions, substantial functional limitations, increased health and other service needs and increased health care costs [11, 12].

To date, no study has specifically investigated asylum-seeking children with medical complexity. The aim of this study was to perform a detailed analysis of the asylum-seeking children with frequent visits, detailing their underlying medical conditions and analyzing care provided.

## Methods

### Study Design, Setting and Study Population

This study is a retrospective cross-sectional study including patients from January 1, 2016 until December 31, 2017. Patients with previous presentations at our institution before January 2015 were excluded from the analysis. Patients were identified using administrative electronic records. Asylum seeking status has been systematically recorded at our institution since January 2016 using the following criteria: (i) referral from one of the reception and processing centres run by the State Secretariat for Migration, (ii) presentation with a referral sheet declaring the patient as asylum-seeking individual or (iii) presentation with an asylum-seeking identity card [8]. For this study we only further analysed patients with frequent visits. There are no standard definitions for frequent visits of health care institutions. We therefore used the standard criteria most commonly used for visits to the emergency department per year [13].

**Fig. 1:**
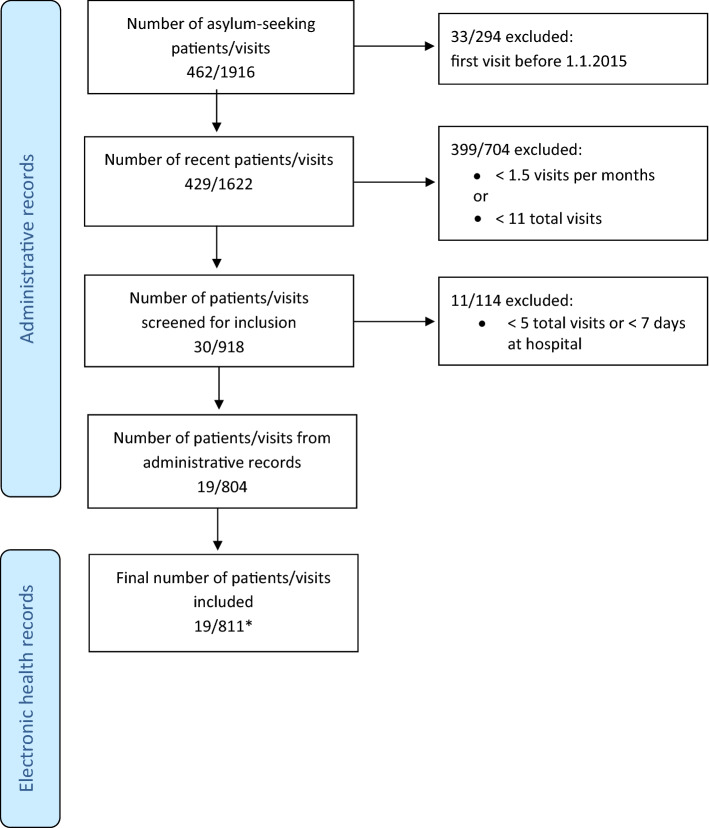
Flowchart depicting the process of inclusion of the study population

In our study period we therefore defined frequent visits as > 10 visits in 24 months. Because some children had only recently arrived, the criteria of ≥ 1.5 visits per month with at least 5 visits or 7 cumulative days of hospital admission was also included (Fig. 1).

### Data Collection and Analysis

Data was extracted from administrative and electronic health records. The following variables were extracted: nationality, age, gender, admission and discharge date, country of birth, escape route, family structure, parental consanguinity, social situation, primary care physician, main diagnoses, vaccination status, and other screenings. Deidentified data was entered in a database (REDCap, Vanderbilt University, 6.9.4). The single data entry was manually reviewed before analysis, REDCap quality control tests were performed, and the records were locked. Matplotlib Python (version 3.0.0, Matplotlib development team) was used for generation of the graphs.

### Definitions

The visiting period was defined as the number of months from the date of the first visit or January 1, 2016 until December 31, 2017. One visit was defined as one consultation from registration to discharge. Consultations by different departments during one hospital admission were therefore not counted as separate visits since the patient was not discharged in between. If a patient was admitted through the emergency department, this was recorded as an additional emergency department visit but not as an additional total visit to ensure that emergency department visits were not lost. Four types of visits were defined: (i) hospital admission, (ii) emergency department visit, (iii) non-physician visit including exercise therapy, occupational therapy, speech therapy and nutritional counseling, (iv) outpatient visit including 15 different outpatient departments.

## Results

A total of 462 asylum-seeking patients with 1916 visits were identified in the 2-year time period. Of those, 19 patients were identified as requiring frequent care, resulting in 811 visits included in the final analysis (Fig. 1).

### Baseline Characteristics of the Study Population

**Fig. 2:**
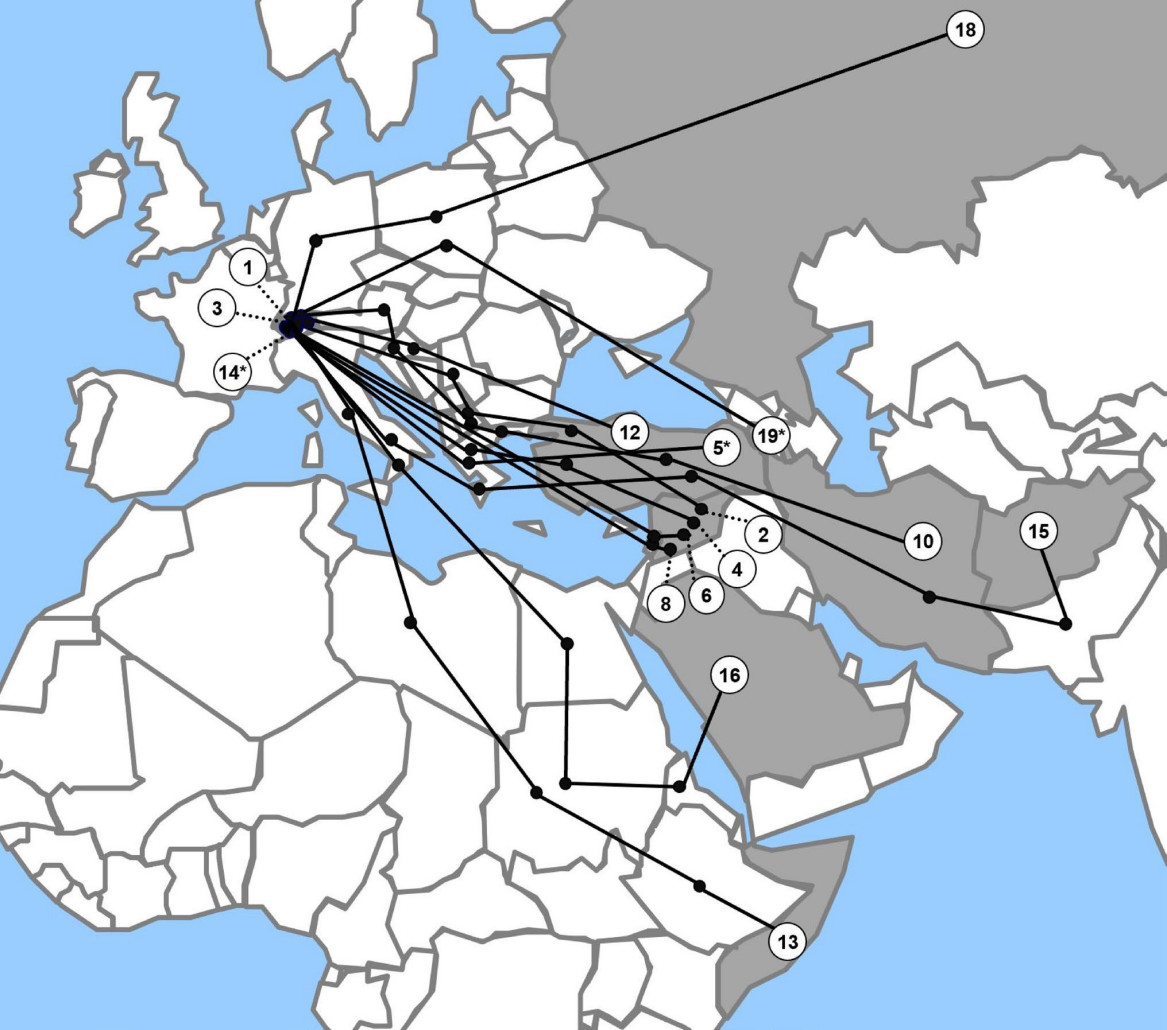
Escape routes (lines) and country of birth (grey) of 16 of the 19 asylum-seeking patients (numbers according to those used in Table 1). Note the route of three patients was not documented. *Denotes the three patients with the highest number of recorded visits

The age range was 0 to 16.7 years with a median of 7 years (IQR 0.4–13.8). Two main age groups were identified: children < 2 years and adolescents > 12 years of age with 8/19 (42%) patients in each group. The patients’ nationalities were Syrian 9/19 (47%), Eritrean 3/19 (16%), Afghani 2/19 (11%), Armenian 2/19 (11%), and Somalian, Algerian and Russian each 1/19 (5%). In eight cases, the country of birth differed from the patient’s nationality and three patients were born in Switzerland. Most patients had prolonged escape routes through several countries (Fig. 2). A total of 10/19 (53%) were accompanied by both parents, 12/19 (63%) by at least one parent, and 3/19 (16%) were unaccompanied minors. The median time between arrival and first consultation at our institution was 3 days. A total of 9/19 (47%) patients visited the hospital within one day after arrival in Switzerland. For four families originating from Syria, Algeria, Russia, and Armenia the reason to leave their home country was the medical condition of their child. At six months after their first visit, 16/19 (84%) of the patients had a primary care pediatrician or general practitioner named in their health record (Table 1).

**Table 1:** Baseline characteristics of 19 asylum-seeking patients

	P1	P2	P3	P4	P5*	P6	P7	P8	P9	P10	P11	P12	P13	P14*	P15	P16	P17	P18	P19*
Gender	f	f	m	m	f	m	m	f	m	m	m	m	m	f	m	m	m	f	m
Time between arrival and first visit (days)	0	153	0	0	1	1	89	0	3	178	3	61	0	0	13	22	366	1	5
Humanitarian visa	–	No	–	Yes	Yes	Yes	Ns	Yes	Ns	No	Ns	Ns	No	–	No	No	No	No	No
Child accompanied by	–	op	–	bp	bp	bp	bp	bp	–	bp	bp	bp	UMR	–	UMR	op	UMR	bp	bp
Distance from last documented address to hospital (km)	1.5	64	16	1.7	1.7	3.7	5.5	1.5	3.7	15	4	18	2.3	0.8	2.4	34	67	4.4	3.5
Total n. of addresses documented	1	1	1	4	4	2	1	3	2	1	1	2	2	2	2	2	2	3	3
Family present in host country	bp	bp	bp	bp	bp	bp	bp	bp	ns	bp	bp	bp	UMR	bp	UMR	bp	UMR	bp	bp
Number of siblings	1	1	3	2	2	1	2	0	ns	2	1	ns	> 5	1	3	> 5	ns	ns	1
Siblings in treatment	Yes	No	Yes	Yes	Yes	No	Yes	No	No	Yes	No	No	No	Yes	No	No	No	No	Yes
Primary care physician	Ped	GP	Ped	Ped	Ped	Ped	Ped	Ped	Ped	Ped	none	Ped	GP	Ped	GP	GP	GP	Ped	none
Hospital social worker documented	Yes	Yes	Yes	Yes	Yes	Yes	No	Yes	No	No	Yes	No	Yes	Yes	Yes	Yes	No	Yes	Yes
Main diagnosis	Noonan-like syndrome	Depression with attempted suicide	Laron-sydrome	Mitochondriopathy	Mitochondriopathy	Marasmus	Failure to thrive of unknown origin	Turner syndrome	Chronic wound infection	Osteochondrosis with chronic pain	Ependymoma	Scalding	Cystic pneumpopathy of uknown origin	Arthrogryposis	Osteomyelitis foot with superinfection	Type 1 diabetes	Severe scoliosis	Complex congenital heart disease	B-cell ALL
ICD-10 of main diagnosis	Q87.1	F32	E34.3	G31.81	G31.81	E41	R62.8	Q96.1	T79.3	M92.5	C72	X19.9	J98.4	Q74.3	M86	E10.1	M41.15	Q21.8	C91.01
Country of first main diagnose	CH	CH	CH	CH	CH	CH	CH	CH	CH	CH	CH	CH	IT	CH	CH	SA	CH	RU	AM

Age, nationality and country of birth are not displayed to protect patient’s identity

*ns* not specified, *op* one parent, *bp* both parents, *UMR* unaccompanied minor refugee, *PED* paediatrician, *GP* general practitioner, *CH* Switzerland, *lT* Italy, *SA* Saudi Arabia, *RU* Russia, *AM* Armenia

*Denotes the three patients with the highest number of recorded visits

### Main Health Problems

**Fig. 3:**
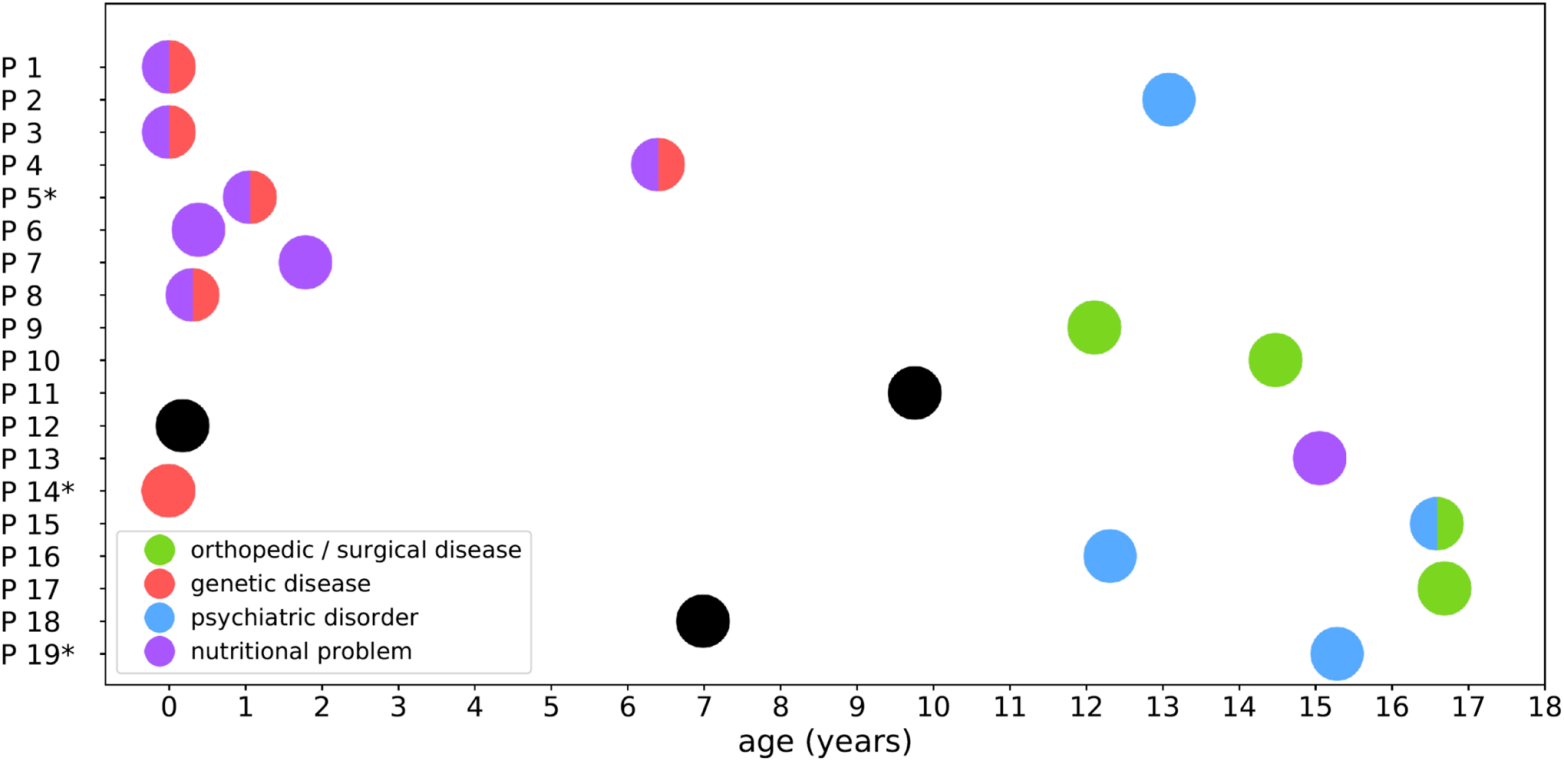
Age distribution (in years) of 19 asylum-seeking patients according to health problems. Note black dots mark patients with health problems not fitting the following categories: orthopedic/surgical disease, genetic disease, psychiatric disorder, nutritional problem. *Denotes the three patients with the highest number of recorded visits

The main health problems and diagnoses are depicted according to age in Fig. 3.

Specifically, in infants 5/8 (63%) had a genetic disease including Noonan-like syndrome (P1), Laron-syndrome (P3), mitochondriopathy (SLC19A3 mutation) (P5), Turner-syndrome (P8), and arthrogryposis (P14). In addition, several associated and non-associated diseases were found including thalassemia minor in one infant (P14). Furthermore 6/8 (75%) had severe malnutrition and feeding problems. Of these, three infants required long-term gastric tube feeding, two infants had percutaneous endoscopic gastrostomy tubes inserted, and two had oral high caloric diet prescribed.

In the adolescent group, 4/8 (50%) had an orthopedic condition or a disease requiring multiple surgical interventions including osteochondrosis (P10), osteomyelitis (P15), severe scoliosis (P17), or chronic wound infection (P9). The main diagnoses in the remaining adolescents were B-cell acute lymphoblastic leukemia (P19), type 1 diabetes (P16), chronic cystic pneumopathy of unknown origin (P13), and depression with attempted suicide (P2). In 3/8 (38%) of the adolescent patients (P15, P16, P19) other psychological conditions were documented including post-traumatic stress disorder and depression. One 15-year-old unaccompanied minor (P13) arrived with severe malnutrition (body mass index of 13 kg/m^2^) and had a percutaneous endoscopic gastrostomy tube inserted. In addition, two of the adolescent patients had diagnoses of latent or active tuberculosis. Three children aged between 2 and 12 years had the following main diagnoses: mitochondriopathy (SLC19A3 mutation) (P 4), ependymoma (P11) and complex congenital heart diseases (P 18) (Table 1).

### Visits by Departments

**Fig. 4:**
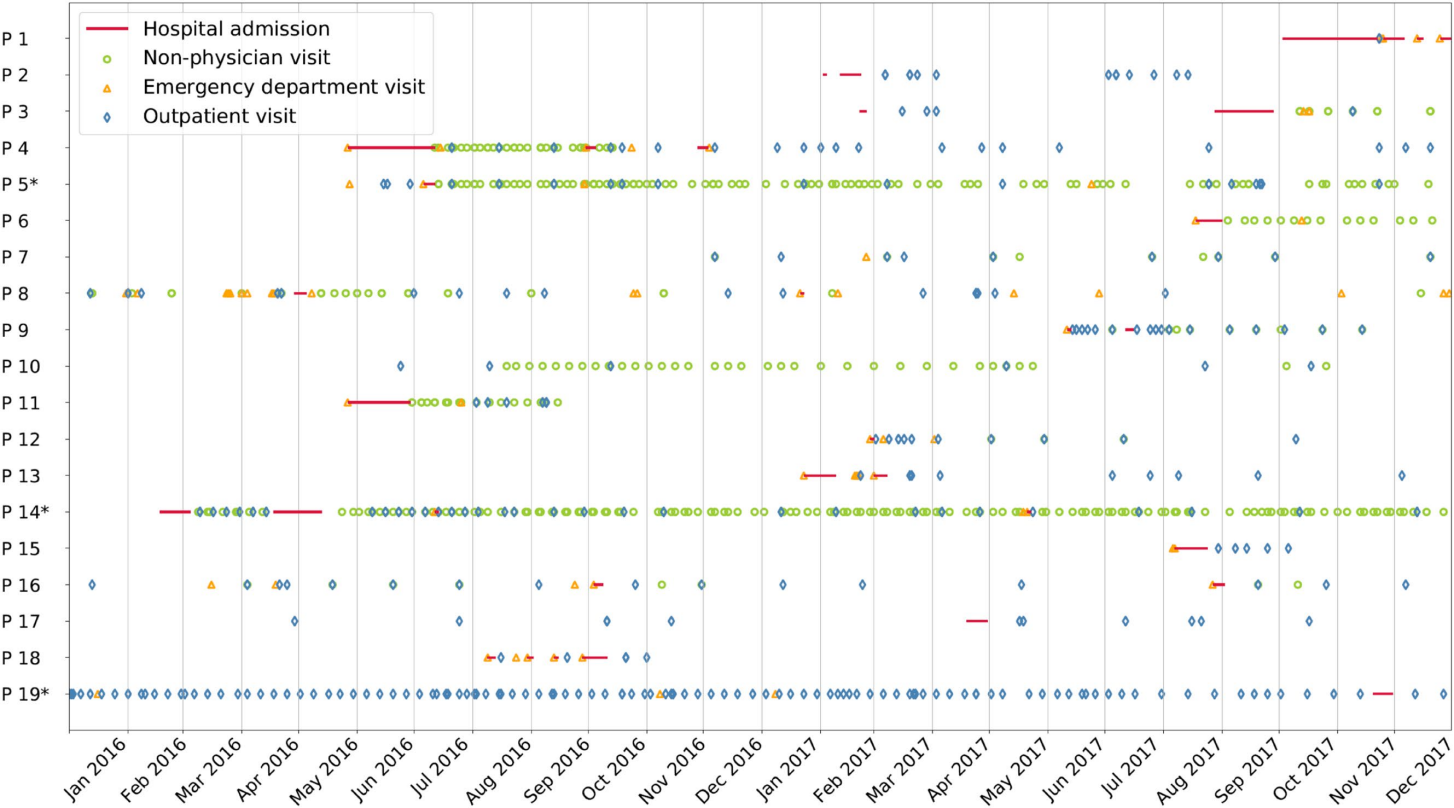
Distribtuion over time of visits of 19 asylum-seeking patients categorised by the main types of visits. Hospital admission (red lines), non-physician visit (green circles), emergency department visit (yellow triangle) and outpatient visit (blue diamond). *Denotes the three patients with the highest number of recorded visits (Color figure online)

There were 811 total visits of which 420/811 (52%) were physician visits. The maximum number of visits per patient were 179 (by P14) and 123 (each by P5 and P19). These three patients accounted for 425/811 (52%) of the total visits (Fig. 4).

Of the physician visits 320/420 (76%) were outpatient visits including the following departments: 123/320 (76%) haemato-oncology by five patients; 47/320 (15%) orthopedics by seven patients, 30/320 (9%) neurology by five patients, and 9/320 (3%) cardiology by five patients. The median number of outpatient visits was 10 (range 0–119). A total of 119/320 (37%) outpatient visits were by P19, a patient with leukemia, visiting the haemato-oncology outpatient department 108 times for treatment on follow up of his disease. Of the physician visits, 66/420 (16%) were at the emergency department. The median number of emergency department visits was 3 (range 0–19) with 3/19 (16%) patients (P2, P10, P17) never requiring a visit.

Admissions accounted for 34/811 (4%) of all visits. The accumulated duration of admission was 350 days with a median duration of 11 (range 0–67) days. Patients had up to four admissions and 10/19 (53%) were admitted more than once.

Non-physician visits accounted for 413/811(51%) of the total visits. These included exercise therapy visits 197/413 (48%) by 6 patients, occupational therapy visits 135/413 (33%) by 6 patients, nutritional counselling visits 46/413 (11%) by 6 patients, and speech therapy visits 35/413 (8%) by 4 patients. Two patients with congenital diseases (mitochondriopathy and arthrogryposis) were responsible for 243/413 (59%) of all non-physician visits. Seven of the 19 (37%) patients never required a non-physician visit.

Infants showed a higher number of total visits (444/811 vs. 267/811), non-physician visits (308/413 vs. 51/413), emergency department visits (38/66 vs. 16/66), a longer duration of hospital admission days (165/ 350 vs. 81/350), and a lower number of outpatient visits (91/320 vs. 196 /320) compared to adolescents.

### Infections and Immunization Status

Screening for methicillin-resistant *Staphylococcus aureus* was done in 9/19 (47%) with two test results being positive (P4, P9). In 9/19 (47%) stool samples were analyzed for infections, of which 5/9 (56%) had a positive result (two each for norovirus and *Hymenolepsis nana* and one each for *Entameoba coli*, *Blastocystis hominis,* and scabies). A total of 5/19 (26%) patients had routine HIV testing without positive results. Screening for tuberculosis was done in two patients, one had a positive result (P13).

An incomplete vaccination status was recorded in 8/19 (42%) patients. In 4/19 (21%) the immunization status was not documented. A total of 3/19 (16%) had been appropriately vaccinated according to the schedule of their home country.

## Discussion

To date, most studies about the health of asylum-seeking children and adolescents have focused on infectious diseases, vaccine preventable diseases, or mental health problems [6, 14, 15]. This the first study analyzing asylum-seeking children with complex and/or chronic illnesses. We found genetic diseases and severe nutritional deficiencies as most prevalent reasons for the frequent need of health care. Importantly, in this group, infectious diseases were rarely the reason for admission and commonly screened infections in asylum seeking and refugee children [16] were only detected in a few patients. Many asylum-seeking children with medical complexity had limited or disrupted access to health care in their countries of origin which negatively impacted the health of the child. For many families, this was an important factor in their decision to escape [9, 17].

Isolated neurodevelopmental disabilities, which are reported to be a major reason for health care visits in children with medical complexity [11], were not seen in our setting investigating the asylum-seeking children with medical complexity. This may be explained by the Swiss health care structure, in which children with neurodevelopmental disabilities, including autism spectrum disorders, are generally cared for in non-hospital based facilities [18]. Alternatively, asylum-seeking families may be less likely to access health care for isolated neurodevelopmental disabilities.

### Prevalence of Diseases

The main diagnoses in the infant group were genetic diseases including rare diseases such as Laron syndrome, a growth hormone insensitivity condition with a prevalence of 1/1,000,000. The occurrence of rare and complex diseases in asylum-seeking children has been described previously but is likely unrecognized. For example, a case series from Germany found six asylum-seeking children with untreated inborn errors of metabolism including phenylketonuria, biotinidase deficiency, HMG-CoA lyase deficiency and mucopolysaccharidoses [9].

Four children in our study originating from Syria had third degree consanguineous parents. Two of them were siblings with a mitochondriopathy and one was a child with Laron syndrome. Autosomal recessive disorders such as inborn errors of metabolism and other genetic diseases are more prevalent in asylum-seeking families from the Middle East, where first-cousin marriages are more common [19]. Whether our findings are applicable for other countries with high number of refugee arrivals is determined by the prevalence of genetic disease in home countries and therefore on the distribution of countries of origin in the respective refugee population.

In our study nutritional problems were frequent amongst the group of infants but also present notably in the adolescent group. This is in line with previous evidence. A study investigating the nutritional status of refugee children in the US detected a 20% higher prevalence of malnutrition and anemia compared to U.S. children [20]. In a large prospective study analyzing 1026 asylum-seeking children at a tertiary care hospital in Australia, nutritional deficiencies were the most common reason for referral [7]. Consequently, specific nutritional treatment programs in refugee camps and an active screening for malnutrition with early interventions and specialty referral are highly recommended [21].

In our study adolescents frequently presented with orthopedic problems and of those who did all needed a surgical procedure. The University Medical Center Mainz described an increase of refugee children being treated for pediatric surgical issues: 12% were in need of medical care due to trauma and 7% due to burns and scalds incurred since their arrival in the host country [21]. In our study population adolescents suffered from chronic orthopedic or surgical problems such as idiopathic scoliosis, osteochondrosis with chronic pain, osteomyelitis, or chronic infection of a laceration.

The results of our study show that psychological assessments were not performed routinely but only when there was a suspicion of a mental disorder. It is therefore possible that psychiatric diseases were underdiagnosed as being a migrant is known to be a risk factor for pediatric and adolescent mental health disorders [22]. Several studies highlight the importance of mental health problems among unaccompanied minor refugees [23–25]. More than 25% of minor refugees develop post-traumatic stress disorder, but only a small proportion of these are diagnosed and treated. The number of suicide attempts among minor asylum-seekers in Europe is higher than in the resident population of the host countries [26]. One 13-year old Syrian girl in our study was admitted due to complications after a suicide attempt. This demonstrates the need for mental health screening to be included in routine medical practice, particularly for unaccompanied minor refugees [25, 27]. Psychosocial and therapeutic treatments should be adapted to the needs of asylum-seeking adolescents in order to be of value and culturally acceptable [28].

There were only few asylum-seeking children with medical complexity aged between 2 and 12 years in our study. This might be due to chance as the study population was limited. Alternatively, it may also be a true finding as infants and children with genetic diseases require frequent hospital admissions in the first few years [29] but once they survive this critical period, less hospital-based visits are required. Furthermore, selection bias might explain the biphasic age distribution. Children with medical complexity below the age of 12 years are unlikely to survive long and exhausting escapes and have a very limited life expectancy particularly in low-income settings [30]. Therefore, those making it to host countries might be the healthier ones among the children with medical complexity, a selection bias, known in the general migrant population as the healthy migrant effect [31]. In addition, the sickest children with medical complexity might have been identified and prioritized by the UNHCR resettlement program according to the current resettlement criteria.

### Frequency of Visits and Departments

The most frequent diagnoses amongst asylum-seeking patients in Germany and Switzerland are respiratory infections [6, 32]. Several studies suggest that asylum-seekers had higher rates of emergency room presentation compared to the resident population [6, 33]. Our study in contrast showed that asylum-seeking children with medical complexity had relatively low emergency department presentation rates and some never required an emergency department visit. A possible explanation might be the close follow up provided by sub-specialized departments to children with medical complexity in Switzerland.

A meta-analysis and a qualitative study on health needs of asylum-seekers from our institution emphasizes the important role of ‘confidence’ between asylum-seeking families and health care providers, demonstrating that it is more likely that families will seek care from a specialist they already know and trust [34]. Furthermore, evidence suggests a lack of continuity of care remains a major problem for asylum-seeking-families [35]. Therefore, coordination between different institutions across countries and across levels of care is crucial [11, 36]. In contrast, most patients in our study had a primary care pediatrician within six months after arrival. This suggests a rapid integration into local health care systems of children with medical complexity in our setting and may offer the possibility of improved integrated care including telemedicine [37].

### Immunization, Screening, and Prevention

In our study a considerable number of patients had an incomplete, or incompletely documented, vaccination status on arrival. Switzerland belongs to one of the countries of the WHO European Region that does not provide free health care to asylum-seeking children and that does not generally include refugees a part of their national immunization program [4]. Per WHO guidance, asylum-seekers should be vaccinated without delay according to the immunization schedules of their host countries if they stay there more than 7 days [38]. Unfortunately, less than one-third of the countries within the WHO European Region focus on the immunization of migrants in their national immunization policies [4, 39].

### Limitations

One potential limitation of this study may be the limited number patients included in the final analysis. However, since the study covered all visits of asylum-seeking patients over two years and aimed to identify patients with frequent visits only, we were not able to influence the sample size. In addition, the limited number of children allowed for the in-depth analysis of over 800 visits. The retrospective study design limited the extent of the data analysis. For the available data there were no missing values for most of the variables, indicating good data quality. Data on cost of care provided was not collected for this analysis, as a health economics analysis was beyond the scope of the current study. Data and information collected by the pediatrician or general practitioner of the patient outside our hospital were not available.

## Conclusion

Asylum-seeking children with medical complexity represent a small but important group of patients requiring frequent medical consultations. The high proportion of young patients with genetic diseases and severe nutritional problems suggests that new strategies are required in the management of this specific group of asylum-seeking children. This could be achieved by exploring options for more integrated peadiatric care via improved coordination between hospital and non-hospital care. In addition, early screening for malnutrition in refugee camps and nutritional treatment programs are needed.

## Electronic supplementary material

Below is the link to the electronic supplementary material.

Supplementary file1 (DOCX 21 kb)

## Abbreviations

B-cell ALL: B-cell acute lymphoblastic leukemia
HIV: Human immunodeficiency virus
UKBB: University Children’s Hospital Basel
UMR: Unaccompanied minor refugee
UNHCR: United Nations High Commissioner for Human Refugees
WHO: World Health Organization
