# Supplementary material for: Asylum-Seeking Children with Medical Complexity and Rare Diseases in a Tertiary Hospital in Switzerland
Source: J Immigr Minor Health. 2020 Oct 20;23(4):669–79. doi: 10.1007/s10903-020-01100-8 (PMC8233290; doi:10.1007/s10903-020-01100-8)
Supplement: Supplementary file 1 — Supplementary file1 (DOCX 21 kb) [file 10903_2020_1100_MOESM1_ESM.docx]

**Supplementary table 1:** Number of visits of 19 asylum-seeking patients per type of visit and department. * denotes the three patients with the highest number of recorded visits

^1^visits leading to hospital admission are included

|  | **P1** | **P2** | **P3** | **P4** | **P5 *** | **P6** | **P7** | **P8** | **P9** | **P10** | **P11** | **P12** | **P13** | **P14*** | **P15** | **P16** | **P17** | **P18** | **P19*** |
| --- | --- | --- | --- | --- | --- | --- | --- | --- | --- | --- | --- | --- | --- | --- | --- | --- | --- | --- | --- |
| ***Vaccination status at arrival*** | 1 | 4 | 1 | 3 | 3 | 3 | 4 | 3 | 3 | 2 | 2 | 1 | 3 | 1 | 3 | 2 | 4 | 3 | 4 |
| *Pychological assessment* | no | yes | no | no | no | no | no | yes | no | no | no | no | no | no | yes | yes | no | no | yes |
| *Neurodevelopmental screening* | yes | yes | yes | yes | yes | no | no | yes | no | no | yes | no | no | yes | no | no | no | no | yes |
| *Dental status* | yes | no | yes | yes | yes | yes | no | yes | no | no | no | no | no | yes | no | no | yes | yes | yes |
| ***Laboratory*** |  |  |  |  |  |  |  |  |  |  |  |  |  |  |  |  |  |  |  |
| *HIV-Testing* | no | no | no | no | no | yes | no | no | no | no | no | no | yes | no | yes | no | yes | no | yes |
| *Result* | - | - | - | - | - | neg | - | - | - | - | - | - | neg | - | neg | - | neg | - | neg |
| *Hepatitis B serology* | no | no | no | no | no | yes | no | no | no | no | no | no | yes | no | yes | no | yes | no | yes |
| *Result* | - | - | - | - | - | neg | - | - | - | - | - | - | pos | - | neg | - | neg | - | neg |
| *Hepatitis C serology* | no | no | no | no | no | no | no | no | no | no | no | no | no | no | no | no | no | no | yes |
| *Result* | - | - | - | - | - | - | - | - | - | - | - | - | - | - | - | - | - | - | neg |
| *Tb-screening* | no | no | no | no | no | no | no | no | no | no | no | no | yes | no | no | no | yes | no | no |
| *Result* | - | - | - | - | - | - | - | - | - | - | - | - | pos | - | - | - | neg | - | - |
| *MRSA-screening* | yes | no | yes | yes | no | no | no | yes | yes | no | no | no | yes | no | yes | no | yes | no | yes |
| *Result* | neg | - | neg | pos | - | - | - | neg | pos | - | - | - | neg | - | neg | - | neg | - | neg |
| ***Microbiology*** |  |  |  |  |  |  |  |  |  |  |  |  |  |  |  |  |  |  |  |
| *Stool sample* | yes | no | yes | no | no | no | yes | yes | no | no | no | no | yes | no | yes | no | yes | yes | yes |
| *Detected pathogen* | Norovirus | - | Norovirus | - | - | - | none | none | - | - | - | - | Blastocystis hominis,  Scabies | - | Hymenolepis nana | - | Hymenolepis nana,Entamoeba coli | none | none |

**Supplementary table 2:** Implementation and results of screenings, clinical examinations and immunization status of 19 asylum-seeking patients.

* denotes the three patients with the highest number of recorded visits

1 = Vaccination schedule according to CH; 2 = Vaccination schedule according to home country; 3 = Incomplete vaccination schedule; 4= Immunization not documented
